# Symptom Monitoring in Ovarian Cancer Patients Treated with PARP Inhibitors: Agreement Between Physician- and Patient-Reported Toxicities Using PRO-CTCAE

**DOI:** 10.3390/cancers18040650

**Published:** 2026-02-17

**Authors:** Roberta Massobrio, Daniela Attianese, Alessandra Testi, Maria Pascotto, Beatrice Campigotto, Claudia Garulli, Luca Pace, Andrea Ricotti, Luca Fuso, Annamaria Ferrero

**Affiliations:** 1 Academic Division of Gynecology and Obstetrics, University of Turin, Azienda Ospedaliera Ordine Mauriziano, 10128 Turin, Italy; roberta.massobrio@unito.it (R.M.);; 2 Clinical Trial Unit, Azienda Ospedaliera Ordine Mauriziano, 10128 Turin, Italy; 3 Department of Surgical Sciences, University of Turin, 10124 Turin, Italy

**Keywords:** ovarian cancer, PRO-CTCAE, PARP inhibitors, toxicities

## Abstract

**Background**: Poly(ADP-ribose) polymerase inhibitor (PARPi) treatment in ovarian cancer patients after first-line chemotherapy and following the response to platinum-based chemotherapy at relapse is associated with survival benefits. Maintenance therapies can be administered over extended periods, making treatment tolerability assessment essential in optimizing patient outcomes. This cohort study aimed to evaluate the agreement between physician and patient reporting of PARP inhibitor-related toxicities and the rate of underestimation of each symptom considered. **Methods**: Patients treated with PARPis in the first-line or recurrent setting were included. A specific Patient-Reported Outcomes Common Terminology Criteria for Adverse Events (PRO-CTCAE) questionnaire was generated and administered to the cohort. For each toxicity, agreement between patients and physicians was assessed using Cohen’s kappa and Gwet’s AC1; in addition, the rate of toxicity under-reporting by physicians was calculated. **Results**: Seventy-seven ovarian cancer patients were included; 39 (50.6%) received PARPis in the first-line setting, while 38 (49.4%) were treated for recurrence. Cohen’s kappa values for agreement between patients and physicians across 12 reported toxicities ranged from 0 to 0.15, indicating poor agreement (κ < 0.20) for all assessed toxicities, with the lowest levels of agreement for decreased appetite (κ = 0), rash (κ = 0.02), headache (κ = 0.00), arthralgia (κ = 0.03), insomnia (κ = 0.03), and fatigue (κ = 0.04). When agreement was assessed using Gwet’s AC1, agreement remained poor to moderate for the majority of the symptoms evaluated. Physician under-reporting rates were higher for nausea (51.9%), rash (57.1%), headache (49.3%), arthralgia (70.2%), insomnia (48.1%), and fatigue (67.5%). **Conclusions**: Our results underscore the importance of systematically integrating patient-reported outcomes into clinical practice, including in maintenance settings, to ensure an accurate assessment of treatment-related toxicities.

## Simple Summary

PARP inhibitors improve survival in ovarian cancer patients after first-line chemotherapy and platinum-sensitive relapse. As maintenance therapies are often prolonged, assessing treatment tolerability is crucial. This cohort study evaluated the agreement between physician- and patient-reported PARPi-related toxicities and identified underestimation rates for each symptom. A total of 77 patients receiving PARPis were enrolled, and a customized PRO-CTCAE questionnaire was used to collect patient-reported toxicities. Of the cohort, 39 received PARPis in first-line maintenance and 38 for recurrence. Agreement between patients and physicians was poor across all 12 toxicities (κ = 0–0.15), with the lowest agreement and highest under-reporting for fatigue, arthralgia, rash, and insomnia. These findings support the routine use of patient-reported outcomes to improve toxicity monitoring in maintenance settings.

## 1. Introduction

Ovarian cancer is a relatively rare disease, representing 1.0% of all new cancer cases in the U.S.; however, in 55% of cases, the diagnosis is determined in the advanced stage, with a 5-year survival rate of 31.5% [1]. Debulking surgery in the absence of residual disease is the main prognostic factor, and chemotherapy with platinum-based combinations represents a milestone in treatment. The mortality rate has slightly decreased in recent decades, reaching 6.3% according to the most recent data, and the introduction of maintenance therapies has certainly changed the treatment outlook for ovarian carcinoma, especially regarding the customization of treatment based on the response to platinum chemotherapy and molecular markers [2,3]. Poly(ADP-ribose) polymerase (PARP) inhibitors (PARPis) have become an important component of the therapeutic armamentarium for ovarian cancer. These agents have demonstrated a survival benefit, particularly in patients with BRCA mutations and in those with homologous recombination-deficient tumors. In addition, their impact on progression-free survival (PFS) in both the first-line and recurrent setting has led to their widespread integration into clinical practice [4,5,6,7,8,9,10,11].

Maintenance therapies, in contrast to traditional chemotherapy, are usually extended over time, making quality of life, tolerability, and sustainability of treatment key factors for patient outcomes. Impairments in quality of life and frequent adverse events may reduce compliance, complicating disease management and potentially impacting survival [12].

In clinical practice, toxicity monitoring primarily relies on clinicians’ observations, which may fail to capture all patient-reported symptoms during treatment [13,14]. This gap highlights the importance of implementing interventions that facilitate the early detection and management of treatment-related adverse events. Patient-reported outcomes (PROs) offer a valuable approach, providing direct accounts of a patient’s condition, unaltered by clinician interpretation, and improving symptom management [15]. The National Cancer Institute (NCI) has developed and tested a tailored system: the Patient-Reported Outcomes version of the Common Terminology Criteria for Adverse Events (PRO-CTCAE), a collection of 124 PRO items selected from the 790 items in the CTCAE, designed to identify the most relevant effects of a specific anticancer treatment [16]. A form builder available on the NCI website allows, through a few simple steps, the construction of specific PRO-CTCAE questionnaires focused on the most relevant toxicities related to the drugs under investigation. Translated into many languages, it explores the frequency, severity, and impact of each adverse event on daily life [17].

Patient-reported outcome analyses from the main phase III trials on PARP inhibitor maintenance therapy primarily used instruments assessing overall quality of life, such as the EORTC QLQ-C30 [18] and the EQ-5D-5L [19], or ovarian cancer-specific symptoms and toxicities, such as the FACT-O [20] and EORTC QLQ-OV28 [21]. However, these tools were not designed to specifically capture PARP inhibitor-related adverse effects.

The aim of this study is the clinical application of a specific PRO-CTCAE questionnaire focused on PARP inhibitor-related toxicities, assessing the agreement between physicians and patients in reporting toxicities and the rate of underestimation of each symptom included in the investigation.

## 2. Materials and Methods

This observational cohort study included ovarian cancer patients treated with PARPis in the first-line or recurrent setting at the Gynecologic Oncology Unit of the Academic Division of Gynecology and Obstetrics, Mauriziano Hospital, Torino, Italy, from April 2021 to December 2024. Clinical and demographic data were collected, including age, performance status, comorbidities, tumor histology, BRCA mutational status and HRD test results if available, PARPi treatment setting (first-line or recurrent), and type of PARPi administered.

A dedicated PRO-CTCAE questionnaire was generated for the specific toxicities of PARPis, using the form builder developed by the Division of Cancer Control and Population Science in the National Cancer Institute at the National Institutes of Health [22]. The core set of toxicities selected for the PRO-CTCAE questionnaire (Appendix A) included dysgeusia, decreased appetite, nausea, vomiting, constipation, diarrhea, cough, rash, headache, arthralgia, insomnia, and fatigue. Five open free-text options were included to allow patients to report additional symptoms that they were experiencing.

The questionnaire was administered during PARP inhibitor maintenance therapy, with each patient completing it once, immediately after a scheduled visit. Completion was independent, treating physicians were blinded to the patients’ responses, and they were not informed of the specific objectives of the study. Treatment-related toxicities identified by the physician during the visit were reported in medical records according to the Common Terminology Criteria for Adverse Events (CTCAE) v. 5.0 classification.

### Statistical Analysis

Before starting this study, agreement between patients and physicians in toxicity reporting was used to estimate the sample size. Assuming a symptom prevalence of 50% for both raters, and defining a clinically relevant difference in Cohen’s kappa of 0.4 (κ 1 = 0, κ 2 = 0.4), with a significance level of 5% and power of 95%, a sample size of 76 patients was calculated.

Descriptive statistics were used to describe demographic and clinical characteristics and PARPi-related toxicities. Agreement between patients and physicians in toxicity reporting was assessed through Cohen’s kappa (κ) calculation, with confidence intervals reported for each symptom. Kappa values between 0.81 and 1 indicate very good agreement, those between 0.61 and 0.80 indicate good agreement, those between 0.41 and 0.60 indicate moderate agreement, those between 0.21 and 0.40 indicate fair agreement, and those <0.20 indicate poor agreement, according to Landis and Koch’s guidelines [23].

Because kappa, as a chance-adjusted measure of agreement between two observers, is affected by the distributions of data and their prevalences across the categories that are used [24], we additionally assessed agreement between patients and physicians using Gwet’s AC1 (first-order agreement coefficient) [25], which has been shown to be less affected by prevalence and marginal probabilities, providing a more stable measure of inter-rater agreement [26]. The level of agreement was interpreted according to the benchmarks proposed by Landis and Koch and applied to the AC1, following Gwet’s recommendations [27].

Under-reporting of toxicities was calculated as the number of cases where a toxicity was reported by patients in the PRO-CTCAE questionnaire but not by physicians. The proportion of under-reporting by physicians was estimated for toxicities of any grade and considering only toxicities reported by patients as moderate, severe, and very severe in the PRO-CTCAE questionnaire.

The analysis was performed using the R software, version 4.5.2.

## 3. Results

### 3.1. Population

A total of 77 patients treated with PARP inhibitors was included in the analysis. As reported in Table 1, the mean age was 63.9 years (range 33–86 years) and the majority of patients (89.6%) had an Eastern Cooperative Oncology Group Performance Status (ECOG PS) of 0, while 10.4% had an ECOG PS of 1.

All included patients underwent genetic testing for the BRCA mutation (gBRCA) and/or tumor testing for the somatic BRCA (sBRCA) mutation/homologous recombination deficiency. In total, 27 patients (35.1%) had a germline BRCA 1-2 mutation (gBRCAm), four patients (5.2%) had somatic BRCA mutations (sBRCAm), and two patients (2.6%) had HRD-positive (BRCA wild-type) tumors. The remaining 44 patients (57.1%) were HRD-negative or unknown (BRCA wild-type).

Regarding PARP inhibitor maintenance treatment, 39 patients (50.6%) received PARP inhibitors in the first-line setting and 38 (49.4%) in the recurrent setting. Moreover, 37 patients were treated with olaparib (48%), 33 (42.9%) with niraparib, and seven patients (9.1%) received rucaparib. Four patients were treated with olaparib despite they were BRCA wild type in the context of clinical trials, i.e., two in the first-line and two in the recurrent setting.

### 3.2. PARP Inhibitor Toxicities

Hematological toxicities were reported across all treatment groups (Table 2).

Anemia was the most frequently observed adverse event: it occurred in 32 patients (86.5%) in the olaparib group, with nine patients (24.3%) reporting grade 3 events; similarly, 29 patients treated with niraparib (87.9%) experienced anemia, with 11 patients (33.3%) with severe toxicity. All seven patients (100%) in the rucaparib group experienced anemia, with one patient (14.3%) reporting a grade ≥ 3 event.

Regarding thrombocytopenia, it occurred in four patients (10.8%) treated with olaparib, seven patients (21.2%) with niraparib, and three patients (42.9%) with rucaparib.

Neutropenia occurred infrequently across all treatment arms: 8.1% in patients treated with olaparib, 9.1% with niraparib, and 14.3% with rucaparib. None of the patients enrolled in this study experienced myelodysplastic syndromes or acute myeloid leukemia.

The non-hematological toxicities declared by patients through PRO compilation and described by physicians were reported and categorized in terms of both all grades and severe/very severe occurrences (Table 3).

Focusing on patient-reported toxicities, fatigue was the most prevalent, affecting 78.4% of patients on olaparib, 87.9% on niraparib, and all patients on rucaparib; severe fatigue occurred in 24.3%, 30.3%, and 43% of patients, respectively. Other commonly reported toxicities included arthralgia (64.9% olaparib, 69.7% niraparib, 29% rucaparib), insomnia (62.2% olaparib, 63.6% niraparib), rash (56.8% olaparib, 54.5% niraparib, 71% rucaparib), nausea (51.4% olaparib, 42.4% niraparib, 71% rucaparib), constipation (35.1% olaparib, 48.5% niraparib, 71% rucaparib), headache (32.4% olaparib, 51.5% niraparib, 29% rucaparib), and dysgeusia (35.1% olaparib, 36.3% niraparib, 43% rucaparib).

Although severe non-hematologic events were less frequent overall, severe and very severe symptoms were especially reported for fatigue (24.3% for olaparib, 30.3% for niraparib, 43% for rucaparib), arthralgia (particularly with niraparib, 21.2%), insomnia (13.5 for olaparib and 15.2 for niraparib), and decreased appetite (13.5% for olaparib, 15.2% for niraparib, 14% for rucaparib).

In the free-text responses, additional reported toxicities included concentration difficulties in two patients (2.3%), hypoacusis or tinnitus in three patients (3.9%), abdominal pain in two patients (2.3%), and vertigo in one patient (1.3%). Regarding these additional symptoms, physicians reported tinnitus in one patient (1.3%), abdominal pain in two patients (2.3%), and vertigo in one patient (1.3%).

### 3.3. Agreement Between Physician- and Patient-Reported Toxicities

A detailed comparison of the toxicity reports by patients and physicians is displayed in Table 4, with the level of agreement between them expressed as Cohen’s kappa and Gwet’s AC1 values.

Across the twelve symptoms reported, the Cohen’s kappa values ranged between 0 and 0.15, indicating poor agreement (κ < 0.20) for all variables considered. The lowest k values were reported for decreased appetite (κ = 0), rash (κ = 0.02), headache (κ = 0.00), arthralgia (κ = 0.03), insomnia (κ = 0.03), and fatigue (κ = 0.04).

When agreement was assessed using Gwet’s AC1, higher levels of agreement were observed; however, for the majority of the symptoms evaluated, the agreement remained poor to moderate.

**Figure 1:**
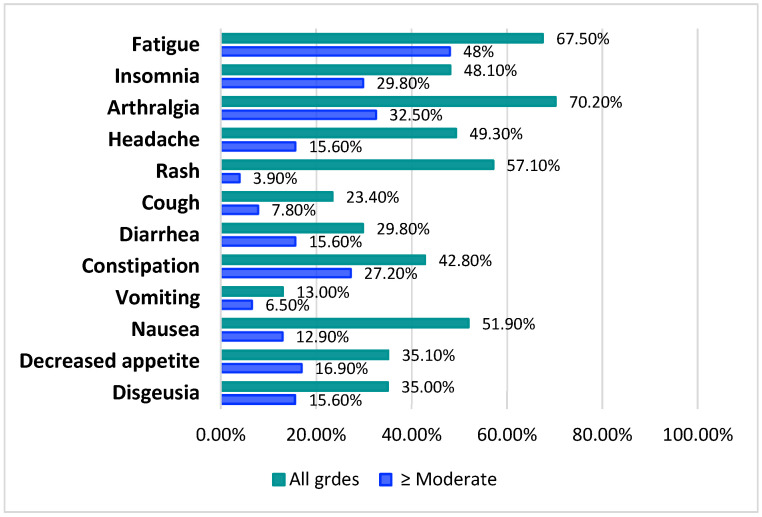
Proportion of under-reporting by physicians for toxicities of any grade (green) and considering only toxicities reported as moderate, severe, and very severe in PRO-CTCAE questionnaire (blue).

For all symptoms analyzed, the percentages of toxicities reported by patients were higher than those reported by physicians. The proportions of under-reporting by physicians (Figure 1), calculated as the number of cases where a toxicity was reported by patients in the PRO-CTCAE questionnaire but not by physicians, were 35% for dysgeusia, 35.1% for decreased appetite, 51.9% for nausea, 13.0% for vomiting, 42.8% for constipation, 29.8% for diarrhea, 23.4% for cough, 57.1% for rash, 49.3% for headache, 70.2% for arthralgia, 48.1% for insomnia, and 67.5% for fatigue.

Excluding the symptoms classified as mild by patients, and therefore considering moderate, severe, and very severe symptoms, the proportions of under-reporting were 15.6 for dysgeusia, 16.9% for decreased appetite, 12.9% for nausea, 6.5% for vomiting, 27.2% for constipation, 15.6% for diarrhea, 7.8% for cough, 3.9% for rash, 15.6% for headache, 32.5% for arthralgia, 29.8% for insomnia, and 48.0% for fatigue.

## 4. Discussion

Our study revealed overall limited agreement between clinicians and patients in their assessments of treatment-related toxicities, with Cohen’s kappa values below 0.20 for all items analyzed and Gwet’s AC1 values indicating poor to moderate agreement for the majority of symptoms. The proportions of under-reporting by physicians were higher (>50%) for fatigue (67.5%), arthralgia (70.2%), rash (57.1%), and nausea (51.9%), and symptoms were often under-reported even when they were classified as moderate, severe, or very severe by patients.

The under-reporting of treatment-related toxicities is well documented, with multiple prior studies demonstrating poor concordance between clinician recognition of symptoms and patient self-reports [13,14,15,16,17,18,19,20,21,22,23,24,25,26,27,28,29].

Bash et al. [14] conducted a prospective analysis comparing symptom occurrence and severity reported by cancer patients and their clinicians, finding low agreement. Interestingly, their study showed a higher level of concordance for adverse effects potentially related to treatment, in contrast to symptoms primarily caused by the disease itself. However, in their study, clinicians were aware that their assessments would be compared with patient reports, which may have contributed to the higher agreement compared to our findings. Consistent with our results, they observed that the agreement between clinicians and patients was higher for symptoms that are more easily observable, such as vomiting and diarrhea. In contrast, more subjective symptoms like fatigue were frequently under-reported by clinicians. Similarly, in previous research in the palliative care setting [28], physical symptoms were more likely to be detected by nurses, while more subjective symptoms were often not reported. These findings align with our results, where symptoms such as insomnia, headache, arthralgia, and fatigue showed the lowest levels of agreement. This is especially important in the context of PARP inhibitor maintenance therapy, where, as demonstrated by both our data and phase III studies, fatigue emerges as a highly prevalent symptom [30]. The recently published NiQoLe study [31] reported physician-assessed adverse events and, as a secondary endpoint, patient-reported symptoms associated with niraparib treatment in relapsed ovarian cancer. The symptoms most frequently reported by patients were fatigue (93%), insomnia (90%), and constipation (86%). Compared with physician-reported toxicities, gastrointestinal symptoms and fatigue were reported up to three times more frequently by patients. Although the data were collected in the specific context of niraparib maintenance therapy for relapsed ovarian cancer and no formal agreement analysis between physician- and patient-reported outcomes was performed, these findings are consistent with our results, highlighting the importance of incorporating PROs in the maintenance treatment setting.

In the context of randomized trials, the accurate reporting of toxicities is critical, as under-reporting can significantly affect toxicity estimates, which are essential for the application of trial findings in clinical practice, particularly when evaluating novel therapies. Based on this rationale, Di Maio et al. [13] analyzed the concordance between patient- and physician-reported toxicities using prospectively collected data from three large randomized trials. Their study focused on six common symptoms associated with anticancer treatments (anorexia, nausea, vomiting, constipation, diarrhea, and alopecia) and revealed substantial discrepancies in reporting toxicities, with frequent under-reporting by physicians. Several factors may contribute to the discrepancies between clinician and patient reports, including inconsistent questioning, time constraints during clinic visits, and attribution bias, where clinicians focus more on anticipated or serious adverse events rather than symptoms that the patient may be experiencing. Additionally, patients may be reluctant to report certain symptoms if they are concerned about treatment dose reductions or discontinuation [32].

The implementation of PROM for symptom self-reporting has been shown to improve patient satisfaction and communication between patients and clinicians, facilitating the earlier detection of serious adverse events [33]. Furthermore, in a recent large randomized trial [34], the use of weekly electronic PRO surveys for symptom monitoring led to significant improvements in physical function, symptom control, and health-related quality of life after 3 months. The authors hypothesized that applying PROM might influence survival outcomes; however, data on the study’s primary endpoint, namely overall survival, are still pending.

Data from randomized phase III trials suggest that PARP inhibitor maintenance therapy does not adversely affect quality of life [35,36]; however, real-world evidence remains limited. The clinical impact of symptom monitoring during PARP inhibitor therapy was not within the scope of our study. Nevertheless, evaluating the influence of PRO implementation on treatment discontinuation, dose modifications, survival, and quality of life represents an important area for future research.

Unlike active treatment phases, maintenance therapies are often extended over time. In the PRIMA [5] and ATHENA-MONO [37] trials, median exposure to PARP inhibitors lasted 11 and 14 months, respectively, while, for patients with BRCA mutations in SOLO1 [10] and SOLO2 [7], the treatment durations were 24.6 and 29.1 months. These extended treatments can be associated with relevant toxicities, which are often poorly tolerated by patients, who are already affected by chemotherapy-related adverse events and expect greater tolerability from an oral maintenance therapy. This can significantly impact quality of life. In this context, the comprehensive evaluation of toxicities and meticulous patient management, incorporating PROs, is essential to optimize tolerability, improve adherence to treatment, and minimize the impact on quality of life.

The strengths of our study include the development of a specific PRO-CTCAE questionnaire tailored to capture PARP inhibitor-related toxicities. Previous studies have frequently utilized quality-of-life questionnaires, such as the EORTC QLQ-C30 [18], which captures both global health status and symptoms. In contrast, our study employed a concise and easy to administer PRO-CTCAE questionnaire, which focused exclusively on treatment-related symptoms. With only 12 items, the questionnaire aligns with current recommendations for the practical application of patient-reported outcome measures in clinical settings [15]. In addition, the integration of five free-text fields allowed patients to report atypical toxicities associated with PARP inhibitor treatment, such as, in our cohort, concentration difficulties, tinnitus, and vertigo. Previous studies have evaluated the agreement between physician- and patient-reported symptoms; however, to the best of our knowledge, none has specifically assessed agreement in the PARP inhibitor maintenance setting using a newly developed, dedicated instrument. In addition, in our study, agreement was evaluated using two different statistical methods, Cohen’s kappa and Gwet’s AC1, and the limited concordance in toxicity recognition was confirmed by both approaches.

A potential limitation of our study, however, is the temporal discrepancy between the patient and clinician reporting periods: the patient-reported symptoms referred to the previous week, whereas clinician-assessed toxicities covered the entire past month, consistent with the monthly clinical visits for patients treated with PARP inhibitors at our center. This discrepancy may explain why, in a few cases, toxicities were reported by physicians but not by patients. Nevertheless, the observed disagreement was mainly driven by toxicities reported through the PRO-CTCAE questionnaire that were not documented by physicians, and patients could have reported even higher rates of symptoms had they reflected on the entire treatment month rather than only the previous week. These findings suggest that physician under-reporting may be more substantial than detected and that the optimal monitoring of treatment-related toxicities may require weekly rather than monthly assessments. Furthermore, the PRO-CTCAE questionnaire was administered at a single time point per patient, as the primary objective of our study was to assess agreement between patients and physicians in toxicity reporting. A longitudinal assessment, however, would provide a more comprehensive evaluation of treatment-related toxicities and potential changes in agreement over time.

## 5. Conclusions

Our results highlight the persistent challenge of capturing patient-reported outcomes, particularly for subjective symptoms that clinicians may underestimate or overlook. This underscores the importance of integrating patient-reported outcomes more systematically into clinical practice, not only during chemotherapy but also in maintenance settings, to ensure a more comprehensive and accurate assessment of treatment-related toxicities and enable more prompt intervention, therefore potentially impacting patients’ quality of life.

## Figures and Tables

**Table 1:** Patients and treatment characteristics.

Characteristic	No.	%
Age	Mean 63.9 (range 33–86)	/
ECOG PS 0	69	89.6%
ECOG PS 1	8	10.4%
gBRCAm	27	35.1%
sBRCAm	4	5.2%
HRD-positive (BRCAwt)	2	2.6%
HRD-negative/unknown (BRCAwt)	44	57.1%
Olaparib	37	48.0%
Niraparib	33	42.9%
Rucaparib	7	9.1%
First-line	39	50.6%
Recurrent setting	38	49.4%

**Table 2:** Hematological toxicities.

	Olaparib (*n* = 37)		Niraparib (*n* = 33)		Rucaparib (*n* = 7)	
	All Grades	G ≥ 3	All Grades	G ≥ 3	All Grades	G ≥ 3
Anemia	32 (86.5%)	9 (24.3%)	29 (87.9%)	11 (33.3%)	7 (100%)	1 (14.3%)
Thrombocytopenia	4 (10.8%)	0	7 (21.2%)	5 (15.2%)	3 (42.9%)	1 (14.3%)
Neutropenia	3 (8.1%)	0	3 (9.1)	1 (3%)	1 (14.3)	0

**Table 3:** Non-hematological toxicities.

	Olaparib (*n* = 37)				Niraparib (*n* = 33)				Rucaparib (*n* = 7)			
	Patient-Reported (PRO-CTCAE)		Physician-Reported (CTCAE)		Patient-Reported (PRO-CTCAE)		Physician-Reported (CTCAE)		Patient-Reported (PRO-CTCAE)		Physician-Reported (CTCAE)	
Symptom	All Grades (%)	Severe/Very Severe (%)	All Grades (%)	≥G3 (%)	All Grades (%)	Severe/Very Severe (%)	All Grades (%)	≥G3 (%)	All Grades (%)	Severe/Very Severe (%)	All Grades (%)	≥G3 (%)
Dysgeusia	13 (35.1)	2 (5.4)	0 (0)	0 (0)	12 (36.3)	2 (6.1)	1 (3)	0 (0)	3 (43)	1 (14)	0 (0)	0 (0)
Decreased appetite	10 (27)	5 (13.5)	0 (0)	0 (0)	13 (39.4)	5 (15.2)	0 (0)	0 (0)	4 (57)	1 (14)	0 (0)	0 (0)
Nausea	19 (51.4)	2 (5.4)	3 (8.1)	1(2.7)	14 (42.4)	1 (3)	3 (9.1)	0 (0)	5 (71)	2 (29)	0 (0)	0 (0)
Vomiting	6 (16.2)	3 (8.1)	0 (0)	0 (0)	4 (12.1)	2 (6.1)	1 (3)	0 (0)	1 (14)	0 (0)	0 (0)	0 (0)
Constipation	13 (35.1)	6 (16.2)	2 (5.4)	0 (0)	16 (48.5)	3 (9.1)	0 (0)	0 (0)	5 (71)	1 (14)	0 (0)	0 (0)
Diarrhea	12 (32.4)	2 (5.4)	1 (2.7)	0 (0)	10 (30.3)	2 (6.1)	1 (3)	0 (0)	1 (14)	0 (0)	0 (0)	0 (0)
Cough	10 (27)	2 (5.4)	0 (0)	0 (0)	6 (18.2)	2 (6.1)	1 (3)	0 (0)	2 (29)	0 (0)	0 (0)	0 (0)
Rash	21 (56.8)	0 (0)	0 (0)	0 (0)	18 (54.5)	1 (3)	1 (3)	0 (0)	5 (71)	1 (14)	0 (0)	0 (0)
Headache	12 (32.4)	2 (5.4)	0 (0)	0 (0)	17 (51.5)	2 (6.1)	2 (6.1)	0 (0)	2 (29)	0 (0)	0 (0)	0 (0)
Arthralgia	24 (64.9)	3 (8.1)	2 (5.4)	0 (0)	23 (69.7)	7 (21.2)	1 (3)	0 (0)	2 (29)	1 (14)	0 (0)	0 (0)
Insomnia	23 (62.2)	5 (13.5)	1 (2.7)	0 (0)	21 (63.6)	5 (15.2)	0 (0)	0 (0)	4 (57)	0 (0)	0 (0)	0 (0)
Fatigue	29 (78.4)	9 (24.3)	5 (13.5)	2 (5.4)	29 (87.9)	10 (30.3)	4 (12.1)	1 (3)	7 (100)	3 (43)	0 (0)	0 (0)

**Table 4:** Agreement between patient and physician reporting of toxicity.

	Toxicity Reported by Neither Patient nor Physician		Toxicity Reported by Physician but Not Patient		Toxicity Reported by Patient but Not Physician		Toxicity Reported by Both Patient and Physician		Agreement Analysis	
Toxicity	No.	%	No.	%	No.	%	No.	%	Cohen’s κ (95% CI)	Gwet’s AC1 (95% CI)
Dysgeusia	49	63.6	0	0	27	35.0	1	1.4	0.05 (0.00 to 0.13)	0.49 (0.34 to 0.65)
Decreased appetite	50	64.9	0	0	27	35.1	0	0	0.00 (0.00 to 0.00)	0.51 (0.36 to 0.66)
Nausea	31	40.3	1	1.3	40	51.9	5	6.5	0.07 (0.00 to 0.16)	0.04 (0.00 to 0.24)
Vomiting	66	85.7	0	0	10	13.0	1	1.3	0.15 (0.00 to 0.40)	0.85 (0.76 to 0.94)
Constipation	42	54.5	0	0	33	42.8	2	2.6	0.06 (0.00 to 0.15)	0.33 (0.15 to 0.50)
Diarrhea	52	67.6	0	0	23	29.8	2	2.6	0.11 (0.00 to 0.24)	0.58 (0.44 to 0.72)
Cough	58	75.3	0	0	18	23.4	1	1.3	0.08 (0.00 to 0.22)	0.70 (0.58 to 0.72)
Rash	32	41.6	0	0	44	57.1	1	1.3	0.02 (0.00 to 0.05)	0.02 (0.00 to 0.21)
Headache	37	48	1	1.3	38	49.3	1	1.4	0.00 (0.00 to 0.07)	0.17 (0.00 to 0.35)
Arthralgia	20	25.9	0	0	54	70.2	3	3.9	0.03 (0.00 to 0.06)	0.00 (0.00 to 0.00)
Insomnia	39	50.6	0	0	37	48.1	1	1.3	0.03 (0.00 to 0.08)	0.23 (0.05 to 0.41)
Fatigue	16	20.8	1	1.3	52	67.5	8	10.4	0.04 (0.00 to 0.11)	0.00 (0.00 to 0.00)

## Data Availability

The data presented in this study are available on request from the corresponding author.

## Appendix A

PRO-CTCAE questionnaires were generated based on the specific toxicities of PARPis using the form builder developed by the Division of Cancer Control and Population Science in the National Cancer Institute at the National Institutes of Health.

NCI PRO-CTCAE ™ ITEMS
As individuals go through treatment for their cancer, they sometimes experience different symptoms and side effects. For each question, please select the one response that best describes your experiences over the past 7 days…

1a. In the last 7 days, what was the SEVERITY of your PROBLEMS WITH TASTING FOOD OR DRINK at their WORST?										
O None	O Mild			O Moderate			O Severe		O Very severe	
				
2a. In the last 7 days, what was the SEVERITY of your DECREASED APPETITE at its WORST?										
O None	O Mild			O Moderate			O Severe		O Very severe	
2b. In the last 7 days, how much did DECREASED APPETITE INTERFERE with your usual or daily activities?										
O Not at all	OA little bit			O Somewhat			O Quite a bit		O Very much	
				
3a. In the last 7 days, how OFTEN did you have NAUSEA?										
O Never	O Rarely			O Occasionally			O Frequently		O Almost constantly	
3b. In the last 7 days, what was the SEVERITY of your NAUSEA at its WORST?										
O None	O Mild			O Moderate			O Severe		O Very severe	
				
4a. In the last 7 days, how OFTEN did you have VOMITING?										
O Never	O Rarely			O Occasionally			O Frequently		O Almost constantly	
4b. In the last 7 days, what was the SEVERITY of your VOMITING at its WORST?										
O None	O Mild			O Moderate			O Severe		O Very severe	
				
5a. In the last 7 days, what was the SEVERITY of your CONSTIPATION at its WORST?										
O None	O Mild			O Moderate			O Severe		O Very severe	
				
6a. In the last 7 days, how OFTEN did you have LOOSE OR WATERY STOOLS (DIARRHEA/DIARRHOEA)?										
O Never	O Rarely			O Occasionally			O Frequently		O Almost constantly	
				
7a. In the last 7 days, what was the SEVERITY of your COUGH at its WORST?										
O None	O Mild			O Moderate			O Severe		O Very severe	
7b. In the last 7 days, how much did COUGH INTERFERE with your usual or daily activities?										
O Not at all	O A little bit			O Somewhat			O Quite a bit		O Very much	
				
8a. In the last 7 days, did you have any RASH?										
O Yes					O No					
	
9a. In the last 7 days, how OFTEN did you have a HEADACHE?										
O Never	O Rarely			O Occasionally			O Frequently		O Almost constantly	
9b. In the last 7 days, what was the SEVERITY of your HEADACHE at its WORST?										
O None	O Mild			O Moderate			O Severe		O Very severe	
9c. In the last 7 days, how much did your HEADACHE INTERFERE with your usual or daily activities?										
O Not at all	O A little bit			O Somewhat			O Quite a bit		O Very much	
				
10a. In the last 7 days, how OFTEN did you have ACHING JOINTS (SUCH AS ELBOWS, KNEES, SHOULDERS)?										
O Never	O Rarely			O Occasionally			O Frequently		O Almost constantly	
10b. In the last 7 days, what was the SEVERITY of your ACHING JOINTS (SUCH AS ELBOWS, KNEES, SHOULDERS) at their WORST?										
O None	O Mild			O Moderate			O Severe		O Very severe	
10c. In the last 7 days, how much did ACHING JOINTS (SUCH AS ELBOWS, KNEES, SHOULDERS) INTERFERE with your usual or daily activities?										
O Not at all	O A little bit			O Somewhat			O Quite a bit		O Very much	
				
11a. In the last 7 days, what was the SEVERITY of your INSOMNIA (INCLUDING DIFFICULTY FALLING ASLEEP, STAYING ASLEEP, OR WAKING UP EARLY) at its WORST?										
O None	O Mild			O Moderate			O Severe		O Very severe	
11b. In the last 7 days, how much did INSOMNIA (INCLUDING DIFFICULTY FALLING ASLEEP, STAYING ASLEEP, OR WAKING UP EARLY) INTERFERE with your usual or daily activities?										
O Not at all	O A little bit			O Somewhat			O Quite a bit		O Very much	
				
12a. In the last 7 days, what was the SEVERITY of your FATIGUE, TIREDNESS, OR LACK OF ENERGY at its WORST?										
O None	O Mild			O Moderate			O Severe		O Very severe	
12b. In the last 7 days, how much did FATIGUE, TIREDNESS, OR LACK OF ENERGY INTERFERE with your usual or daily activities?										
O Not at all	O A little bit			O Somewhat			O Quite a bit		O Very much	
				
OTHER SYMPTOMS										
Do you have any other symptoms that you wish to report?										
O Yes						O No				
Please list any other symptoms:										
1.		In the last 7 days, what was the SEVERITY of this symptom at its WORST?								
		O None	O Mild			O Moderate		O Severe		O Very Severe
2.		In the last 7 days, what was the SEVERITY of this symptom at its WORST?								
		O None	O Mild			O Moderate		O Severe		O Very Severe
3.		In the last 7 days, what was the SEVERITY of this symptom at its WORST?								
		O None	O Mild			O Moderate		O Severe		O Very Severe
4.		In the last 7 days, what was the SEVERITY of this symptom at its WORST?								
		O None	O Mild			O Moderate		O Severe		O Very Severe
5.		In the last 7 days, what was the SEVERITY of this symptom at its WORST?								
		O None	O Mild			O Moderate		O Severe		O Very Severe
